# Dating violence and mental health issues in Ecuadorian university students

**DOI:** 10.3389/fsoc.2026.1743049

**Published:** 2026-05-11

**Authors:** Mayra Castillo-Gonzáles, Emilio Terán-Andrade

**Affiliations:** 1 Department of Psychology, Faculty of Health Sciences, Sek International University, Quito, Ecuador; 2 School of Law, Universidad de las Americas (UDLA), Quito, Ecuador

**Keywords:** dating violence, mental health, gender differences, university students, young adults

## Abstract

**Objective:**

The aim of this study was to analyze the relationship between dating violence and mental health issues in young university students in Ecuador examining differences between men and women.

**Methods:**

This investigation used a quantitative, descriptive and explanatory approach, cross-sectional design with a sample of 1,033 students. Two psychological scales were applied: Dating Violence Questionnaire-R (DVQ-R) and General Health Questionnaire-28 (GHQ-28). Statistical analyses included chi-square tests, Pearson’s correlation coefficient and multivariate analysis of variance (MANOVA).

**Results:**

Revealed significant differences (*p* < 0.01) in the prevalence of dating violence, which was slightly higher in men, while women scored higher on mental health symptoms. In addition, it was observed that dating violence was significantly associated (*p* < 0.01) with poorer mental health, being associated of psychosomatic symptoms, anxiety, depression and social dysfunction, especially in woman.

**Conclusion:**

This study found that dating violence represents a significant issue affecting both women and men. It is essential to adopt an inclusive and gender-sensitive approach to dating violence regardless of gender.

## 1 Introduction

### 1.1 Dating violence

Dating violence (DaV) is a critical issue with significant implications for both physical and mental health, which is why it is recognized as a major public health concern (Taquette et al., 2019). According to the World Health Organization (2002), intimate partner violence is defined as *“any behavior within an intimate relationship that causes physical, psychological, or sexual harm to those in the relationship”* (p. 97). The WHO highlights that partner violence can manifest in multiple forms, including physical and psychological abuse, coercive control, and sexual violence. However, intimate partner violence has different types, including marital, cohabiting, conjugal and DaV, which represents a specific manifestation occurring typically among adolescents and young populations (Breiding et al., 2015).

In contrast to adult partnerships, violent relationships among young people characteristically do not include economic dependence, shared domestic responsibilities, or co-parenting dynamics (Ramos Rangel, 2024). In this context, abusive behaviors may be normalized or minimized, hindering their recognition. This is particularly relevant given that psychological violence is the most frequently reported in DaV, yet it remains the least visible and often underrecognized. Young people perceive behaviors such as teasing, jealousy, or controlling actions as normative components of romantic relationships (Gracia-Leiva et al., 2019). Also, DaV is often characterized by reciprocal dynamics, in which both partners may act as victims and perpetrators within the same relationship. This bidirectional perspective has gained increasing attention in recent years (Castillo-Gonzáles and Terán Andrade, 2024; Queiroz Ribeiro et al., 2024; Rojas-Solís and Romero-Méndez, 2023), as it highlights the complexity of violent interactions among young couples. Beyond its conceptual complexity, DaV also represents a highly prevalent phenomenon.

The prevalence of DaV has gained increasing attention in recent years and appears to be on the rise (Gracia-Leiva et al., 2019). Recent systematic review and meta-analysis (Queiroz Ribeiro et al., 2024) reported that adolescents and young adults experience levels of DaV, with psychological violence being the most prevalent, followed by physical and sexual forms. Evidence also indicates gender-related patterns, with women more likely to report victimization, while men more frequently report perpetration. These trends are consistent with findings from studies conducted in both African (Johnson et al., 2024) and European populations (Tomaszewska and Schuster, 2021), where higher rates of victimization have been observed among women.

In Ecuador, studies also suggest that women report higher levels of victimization in partner violence (Capelo-Gálvez et al., 2022; IINEC, 2019). However, other studies has reported high levels of DaV affecting both women and men (Castillo-Gonzáles et al., 2024; Castillo-Gonzáles and Terán Andrade, 2024). For example, Castillo-Gonzáles et al. (2024) found that men tended to report higher frequencies of milder forms of psychological, physical, sexual, and instrumental violence, whereas women report greater involvement in more severe forms, particularly in instrumental and psychological victimization. At moderate levels, psychological violence is more frequently reported by men, while women report higher levels of sexual violence. Additionally, evidence indicates the presence of bidirectional patterns of violence, in which both women and men are involved in DaV, where aggressive and controlling behaviors are commonly reported (Castillo-Gonzáles and Terán Andrade, 2024).

Moreover, in Ecuador, traditional gender roles and cultural norms shape the way DaV manifests and interpersonal dynamics that may be associated with the persistence of DaV as reflected in the high rates reported in intimate relationships (Cuadrado-Gordillo and Martín-Mora-Parra, 2022). Previous research has highlighted the role of factors such as hostile sexism, family dynamics, and sociocultural expectations, which may contribute to the persistence and normalization of violent behaviors among young couples (Díaz-Sánchez et al., 2025).

DaV is also associated with mental health issues. Studies (Castillo-Gonzáles et al., 2026; Matud et al., 2023; Muñoz-Rivas et al., 2007; Taquette et al., 2019) worldwide have reported a wide range of psychological problems, as well as mental disorders associated with experiences of abuse in romantic relationships. In this regard, the following section examines in greater depth the relationship between DaV and mental health issues.

### 1.2 DaV and mental health issues

DaV has been widely associated with a variety of adverse mental health (MH) outcomes among adolescents and young adults. Exposure to violence within romantic relationships has been linked to emotional distress, including symptoms of depression, anxiety, post-traumatic stress disorder, eating disorders, addictions, and other internalizing problems (Castillo-Gonzáles et al., 2026; Chen et al., 2025; Kirkman et al., 2025; Lorenzo González et al., 2018; Redondo Pacheco et al., 2022; Tarriño-Concejero et al., 2023).

Both men and women can experience DaV and suffer negative health consequences. Coker et al. (2002) found that young victims of violence, regardless of gender, report somatic symptoms like weight changes, stomach issues, headaches, and nervousness, along with behavioral difficulties such as defiance. The frequency, severity, and duration of violence are associated with increased psychological distress in both sexes. A review by Rojas-Solís et al. (2019) highlighted that male victims may face various mental health issues, including depression, suicidal thoughts, and psychosomatic symptoms. Psychological violence experienced by men may also be diverse and have significant psychological impact. However, studies by Lorenzo González et al. (2018) and Kaura and Lohman (2007) indicate that women are more severely affected, showing higher levels of depression, anxiety, and low self-esteem following intimate partner violence.

Despite the growing body of literature examining DaV and mental health issues, integrating these variables within a comprehensive framework remains limited, particularly in relation to gender differences. Although previous research (Albert, 2015; Burris et al., 2024; Navarro and Carhualla, 2025; Redondo Pacheco et al., 2022; Tarriño-Concejero et al., 2023) has often focused on women due to their higher risk of severe victimization and adverse outcomes, other studies (Betancourt Ocampo et al., 2022; Castillo-Gonzáles et al., 2026; Coker et al., 2002; Kaura and Lohman, 2007; Matud et al., 2023; Rojas-Solís et al., 2019) suggest that both men and women may report comparable levels of victimization in certain contexts. However, it remains unclear how these experiences are differentially associated with mental health problems across genders, particularly in populations within Latin American contexts.

Additionally, in Ecuador, few studies have addressed DaV, and there is a notable lack of research that explores its relationship with MH in depth. Understanding these aspects is essential for the development of public policies, prevention programs, and psychological interventions that address the mental health needs of both men and women. Likewise, understanding this dynamic will contribute to the promotion of gender equality and the well-being of women in relationships. Therefore, the objective of this research is to analyze the relationship between dating violence and mental health issues in young university students in Ecuador, examining differences between men and women. It is hypothesized that dating violence will be significantly associated with mental health outcomes, and that this association will differ between men and women within a multivariate framework.

## 2 Materials and methods

This research adopts a quantitative approach based on the statistical analysis of numerical data. It follows a descriptive and correlational design aimed at examining the relationship between DaV and mental health at a specific point in time. A cross-sectional design was employed, given that the data were collected at a specific time interval. Additionally, the study can be classified as field research, since standardized psychological instruments were administered as part of the data collection process.

### 2.1 Participants

The study population consisted of 32.061 students from two public universities in Ecuador: Universidad Nacional de Chimborazo and Escuela Superior Politécnica de Chimborazo. Data for the present study were collected in July 2024 as part of a broader research project that began in October 2022. The present analysis is based on a specific subsample selected for this study, ensuring the use of consistent measurement procedures. The sample was determined by using the finite population equation, obtaining a sample of 1,033 students. In total, 48.8% of the students were male, and 52.2% were female. Students with a partner numbered 49.1, and 50.9% did not have a partner at the time but had been in a relationship for at least 1 month and all participants were single. The age range was 18–35 years (*M* = 22.61, SD = 4.68). In this study, “men” and “women” refer to young adult university students. Most participants were from middle socioeconomic backgrounds (86.5%). Geographically, 85% of the participants were from urban areas, indicating a higher representation from urban settings. Most ethnically identified as mestizos (90.7%), consistent with national demographics.

### 2.2 Sampling procedure

The sample size was calculated using the finite population formula to ensure statistical representativeness, considering a total population of 32,061 enrolled students, a *Z* value of 1.960 for a 95% confidence level, an assumed proportion (p) of 0.5 to maximize the sample size, and a margin of error (e) of 0.03. A sampling strategy based on the total student population was implemented to guide participant recruitment. The initial step involved contacting faculty deans at the selected universities to explain the aims of the study and request their collaboration. Subsequently, course secretaries provided the institutional email addresses of students. Eligible participants received a formal invitation that included information about the study objectives, voluntary participation, and a link to the anonymous online questionnaire. Participation was voluntary, and therefore, the final sample may be subject to self-selection bias. Informed consent was obtained from all participants prior to participation.

### 2.3 Instruments

*Dating Violence Questionnaire-R (DVQ-R)* is an instrument designed to measure victimization in young people within their romantic relationships. It was validated for Spanish speaking populations by Rodríguez-Díaz et al. (2017). The instrument consists of 20 Likert type items, each offering five response options (0 = “never” to 4 = “very frequently”). These 20 items are grouped into five types of violence: detachment, humiliation, sexual, coercion and physical, and can be categorized as: not present, mild, moderate, or severe. To assess detachment, an example item is: “Does not acknowledge any responsibility regarding the relationship or what happens to both of you?” For humiliation: “Humiliates you in public?” For sexual: “Do they insist on touching you in ways that feel uncomfortable or that you do not want?” For coercion: ““Tests” your love, setting traps to find out if you are cheating?” And for physical: “Has hurt you with an object?.” The overall reliability of the questionnaire was high (*α* = 0.95), with subscale reliabilities as follows: detachment violence (*α* = 0.97), coercion violence (*α* = 0.77), physical violence (*α* = 0.88), sexual violence (*α* = 0.85), and humiliation violence (*α* = 0.87).

*The Goldberg General Health Questionnaire (GHQ-28)* is a self-report instrument used to identify mental disorders. It is a shortened version of the original GHQ, developed by David Goldberg in 1972. The questionnaire consists of 28 items divided into four subscales, each comprising seven questions that assess somatic symptoms (physical symptoms and bodily concerns related to stress and anxiety), anxiety/insomnia (levels of anxiety and sleep-related issues), social dysfunction (the individual’s ability to carry out daily activities and fulfill social roles), and severe depression (symptoms of de-pression and thoughts of self-harm). Each item is rated on a four-point Likert scale ranging from “better than usual” to “much worse than usual.” Scoring methods may vary, but the most commonly used is the binary scoring method (0–0–1–1), which identifies the presence of symptoms. The cutoff point for instrument is 5/6 (no case/case). The GHQ-28 was validated for the Ecuadorian population by Moreta-Herrera et al. (2021) demonstrating its suitability for measuring mental health. The total scale showed excellent reliability (*α* = 0.99), with strong psychometric properties reported in their study for the overall scale (*α* = 0.92) and its sub-scales: somatic symptoms (*α* = 0.77), anxiety (*α* = 0.83), social dysfunction (*α* = 0.79), and depression (*α* = 0.88).

### 2.4 Data quality assurance

Various measures were implemented to ensure the quality and reliability of the data obtained through online surveys, including the presentation of clear informed consent. The exclusion of rapid responses to avoid unreliable data, mechanisms to prevent the submission of incomplete surveys, a pilot test to verify the clarity of the questions. These actions sought to reduce bias and improve the validity of the results.

### 2.5 Data analysis

The data collected were analyzed using IBM SPSS Statistics software, version 25. First, basic descriptive statistics were calculated, including the reliability of scales and subscales using Cronbach’s alpha, normality indices through the Kolmogorov Smirnov test, as well as means and standard deviations. To examine differences in DaV and MH by gender, Chi-square tests and Student’s t-tests were conducted. Pearson’s correlation coefficient was used to assess the relationships between the variables. Additionally, a multivariate analysis of variance *(MANOVA)* was conducted to examine differences in dating violence *(DVQ-R scores)* as a function of mental health dimensions *(GHQ-28)* and gender. This method was chosen to analyze multiple dependent variables simultaneously, allowing for a more comprehensive assessment while accounting for their intercorrelations.

## 3 Results

Percentages of research participants who reported having experienced or not to have experienced overall dating violence (DaV Global, DVQ-R) according to gender are presented. With regard to overall violence, 50.7% of the 501 male participants and 49.3% of the 529 female participants stated that they had suffered violence. The Chi2 test and Bonferroni indicated that there were significant differences (*χ*^2^(1) = 19.9, *p* = 0.005, *V* = 0.14 (small effect size)) in overall violence between men and women. Taking into account the intensity (mild, moderate, or severe) and the types of violence, significant differences were found between men and women (*p* < 0.001) in detachment, humiliation, sexual, coercion and physical violence. Men reported significantly higher levels of global and all types of violence (*p* ≤ 0.05), representative a higher reported prevalence among men (Table 1). Though men reported higher overall prevalence, women showed higher scores in specific and more severe forms of violence.

**Table 1:** Frequency of dating violence among university students by gender.

Types of violence n: 1,033		Men		Women		Chi 2	Sig.
		*n*	*%*	*n*	*%*		
Detachment	No	143a	28.5	174a	32.9	19.4	0.000
	Mild	166a	33.1	196a	37.1		
	Moderate	160a	31.9	127b	24.0		
	Severe	32a	6.4	32a	6.0		
Humiliation	No	260a	51.9	314b	59.4	11.94	0.000
	Mild	101a	20.2	115a	21.7		
	Moderate	109a	21.8	79b	14.9		
	Severe	31a	6.2	21a	4.0		
Physical	No	263a	52.5	365b	69.0	30.0	0.000
	Mild	111a	22.2	79b	14.9		
	Moderate	98a	19.6	69b	13.0		
	Severe	29a	5.8	16b	3.0		
Sexual	No	238a	47.5	320b	60.5	17.8	0.000
	Mild	131a	26.1	98b	18.5		
	Moderate	110a	22.0	92a	17.4		
	Severe	22a	4.4	19a	3.6		
Coercion	No	145a	28.9	214b	40.5	20.9	0.000
	Mild	163a	32.5	172a	32.5		
	Moderate	159a	31.7	123b	23.3		
	Severe	34a	6.8	20b	3.8		

Chi^2^ = chi square; *p* = significance; a-b = Bonferroni method.

As shown in Table 2, there are statistically significant gender differences in mental health, with women scoring higher on all mental health scales—somatic symptoms, anxiety, social dysfunction, and depression. This indicates that women report more mental health problems than men (*t* = −5.10, *p* < 0.001, indicating a moderate effect size).

**Table 2:** Comparative analysis of mental health issues by gender.

Mental health	Men		Women			
	*M*	*SD*	*M*	*SD*	*t*	*p*
Psychosomatic symptoms	12.9	3.8	14.4	4.0	−6.70	<0.001
Anxiety	13.4	4.8	15.1	5.0	−6.00	<0.001
Social Dysfunction	13.9	4.1	14.9	4.0	−4.40	<0.001
Depression	11.3	4.9	11.8	5.2	−1.90	<0.001
Global	51.6	14.6	56.4	15.2	−5.10	<0.001

SD = standard deviation; *t* = Student’s *t*-test; *p* < 0.001.

As shown in Table 3, global DaV is significantly but weakly correlated with mental health, specifically with the psychosomatic subscale (*r* = 0.256, *p* < 0.001), anxiety subscale (*r* = 0.257, *p* < 0.001), social dysfunction subscale (*r* = 0.238, *p* < 0.001), and depression subscale (*r* = 0.363, *p* < 0.001). Additionally, specific types of DaV are also correlated with the mental health subscales. The findings indicate weak to moderate associations between DaV and mental health dimensions.

**Table 3:** Relationship between DaV and mental health issues.

DVQ-R	GHQ 28			
	Somatic	Anxiety	Social dysfunction	Depression
Detachment	0.309**	0.312**	0.251**	0.359**
Humiliation	0.207**	0.215**	0.205**	0.360**
Physical	0.159**	0.154**	0.153**	0.292**
Sexual	0.195**	0.204**	0.198**	0.352**
Coercion	0.245**	0.254**	0.218**	0.332**
Global	0.256**	0.257**	0.238**	0.363**

**p < 0.001; *p > 0.005.

Multivariate comparisons were conducted for the total scores and the four dimensions of the DVQ-R according to mental health issues (no/yes) and sex, and their interaction (Table 4). The MANOVA revealed a significant major effect of mental health (Wilks’ *λ* = 0.763, *F*(4, 2,291) = 309.264, *p* < 0.001, *η*^2^ = 0.515, indicating a large effect size), sex (Wilks’ λ = 0.972, *F*(6, 2,383) = 4.343, *p* < 0.001, *η*^2^ = 0.020, indicating a small effect size), and the interaction between mental health and sex (Wilks’ λ = 0.975, *F*(6, 2,183) = 2.134, *p* = 0.036, *η*^2^ = 0.005, indicating a small effect size).

**Table 4:** Univariate contrasts and descriptives for dating violence according to mental health issues (No/Yes) and gender.

Dating violence	Sex	Mental health				Total		Univariate contrasts		
		No		Yes						
		*M*	*DT*	*M*	*DT*	*M*	*DT*	*F*	*p*	*η*
Global	Men	41.16	10.69	51.18	14.49	56.19	45.23	54.085	0.000	0.040
	Women	47.94	13.24	56.01	15.16	56.52	45.74			
	Total	45.20	12.69	53.60	15.17	56.40	45.55	1,196.979	0.000	0.272
Detachment	Men	42.76	12.46	52.19	14.27	49.50	14.41	15.566	0.000	0.009
	Women	48.17	12.69	57.12	15.69	54.17	15.35			
	Total	45.73	12.85	54.65	15.19	51.90	15.08	65.419	0.000	0.020
Humiliation	Men	45.49	12.26	53.83	15.30	49.50	14.41	11.766	0.000	0.010
	Women	50.51	14.37	59.52	15.20	54.17	15.35			
	Total	48.24	13.67	56.51	15.50	51.90	15.08	177.219	0.000	0.053
Physical	Men	46.08	12.75	53.28	15.20	49.50	14.41	6,246	0.000	0.005
	Women	52.06	14.90	58.88	15.32	54.17	15.35			
	Total	49.56	14.34	55.56	15.48	51.90	15.08	63.507	0.000	0.019
Sexual	Men	45.80	12.28	52.69	15.34	49.50	14.41	11.859	0.000	0.052
	Women	50.34	14.25	59.91	15.18	54.17	15.35			
	Total	48.42	13.63	55.87	15.67	49.50	15.08	1,311.103	0.000	0.291
Coercion	Men	43.85	12.22	51.80	14.61	49.50	15.35	11.758	0.000	0.030
	Women	49.55	14.73	57.32	14.98	54.17	15.08			
	Total	47.25	14.04	54.39	15.03	51.90	14.41	1,311.103	0.000	0.291

With regard to mental health, the univariate contrasts indicated that study participants with mental health outcomes obtained significantly higher scores (*p* < 0.001) in the total score and all dimensions of the scale, with a large effect on sexual and coercion violence, a medium one on humiliation violence, and a small one on physical and detachment violence. With regard to gender, the univariate contrasts showed significant differences in the total score (*p* ≤ 0.001), that women obtained higher scores in the dimensions of coercion, sexual, and humiliation violence, indicating greater levels of reported violence in these dimensions among women. Participants who report DaV also report poorer mental health issues, with this pattern being more pronounced in women than in men, especially in sexual and coercion violence, which showed the highest effect sizes (*η*^2^ = 0.291, indicating a large effect size) in both cases.

## 4 Discussion

DaV represents a serious problem that requires a deep and multidimensional analysis. This violence can take many forms, including detachment, humiliation, sexual, coercion and physical violence (Rodríguez-Díaz et al., 2017). The results of this study provide information about the relationship between DaV and MH outcomes supporting the proposed hypothesis.

### 4.1 DaV in women and men

A high frequency of DaV was found in both men and women, with a slight increase among men. These results are consistent with studies conducted in different contexts (Alegría Del Ángel and Rodríguez Barraza, 2015; López-Barranco et al., 2022; Matud et al., 2023; Rojas-Solís et al., 2019; Rubio-Garay et al., 2017), which have determined that DaV is a problem that negatively affects both men and women. Some studies even conclude that men are more often victims of psychological, physical and sexual violence in relationships (Castillo-Gonzáles et al., 2024; Díaz Burgos et al., 2023; Pacheco Maldonado and Castañeda Figueroa, 2017; Peña-Cárdenas et al., 2013; Warthe et al., 2025). Scott-Storey et al. (2023) argue that a high percentage of men have been victims of violence within their relationships. Similarly, Watkins et al. (2014) found that although women are more frequently victims of violence, DaV among men is significant and warrants attention.

These findings should be interpreted with caution, prevalence does not necessarily reflect severity or impact, particularly within a bidirectional pattern in which both partners may be involved. As noted in the introduction, several studies in the Ecuadorian context have traditionally emphasized women as victims and men as perpetrators, often overlooking male victimization (Capelo-Gálvez et al., 2022; Díaz-Sánchez et al., 2025; IINEC, 2019). However, the present results support a more complex perspective, indicating that both men and women may experience and report violence within romantic relationships. This aligns with findings from Latin American studies that have reported similar patterns (Alegría Del Ángel and Rodríguez Barraza, 2015; Pacheco Maldonado and Castañeda Figueroa, 2017; Rojas-Solís et al., 2019; Rojas-Solís and Romero-Méndez, 2023). While it is important to acknowledge that women have historically been in more vulnerable positions, recent evidence suggests that bidirectionality is one of the most frequently reported patterns in DaV among young populations.

In Ecuador, gender norms and traditional roles persist (Cuadrado-Gordillo and Martín-Mora-Parra, 2022), which can restrict men’s ability to express emotions. These norms may also contribute to the normalization of violent acts. Although research addressing this specific issue in the country is limited, existing results align with the findings of Castillo-Gonzáles et al. (2024). Their study, based on a sample of university students, also identified high levels of violence reported by both men and women, noting a slightly higher prevalence among men.

### 4.2 MH in women and men

This study found that women experience more MH issues, such as depression, anxiety, psychosomatic symptoms, and social dysfunction (Albert, 2015; Zender and Olshansky, 2009). These results are consistent with studies that reported women experience more MH problems related to anxiety and depression (Burris et al., 2024; Hantsoo and Epperson, 2017). Additionally, McLean et al. (2011) found that most women exhibited symptoms of severe anxiety, and nearly half showed signs of moderate depression, highlighting the significant prevalence of these disorders among women. Therefore, the findings of the present study are consistent with previous research indicating that women report higher levels of mental health problems. Previous research (Baader et al., 2014; Bezerra et al., 2021; Micin and Bagladi, 2011; Teixeira et al., 2022) indicates that MH difficulties are more prevalent among women, particularly in the form of anxiety, depression, psychosomatic symptoms, and behavioral disorders. These issues may also extend to more severe conditions, such as eating disorders, post-traumatic stress, and suicidal tendencies, especially in the presence of interpersonal conflicts. Overall, the literature consistently highlights gender differences in mental health, with women reporting higher levels of internalizing symptoms compared to men. Finally, social dysfunction is a common but often overlooked outcome, especially among women exposed to violent relationships or family conflict, potentially leading to social isolation (Orben et al., 2020; Skoog Waller and Forinder, 2026).

These findings do not imply that men do not experience mental health problems; rather, both men and women are affected. However, existing evidence suggests that women are more likely to report internalizing disorders, which may be associated with a combination of biological, psychological, and social factors (Burris et al., 2024; Hantsoo and Epperson, 2017). In this regard, gender may play a relevant role, as men may be less likely to externalize or express their emotions, potentially leading to reduced reporting of internalizing symptoms. From a sociocultural perspective, particularly within the Ecuadorian context, men are often socialized to adopt roles characterized by strength, dominance, and emotional restraint (Ayol-Gusñay and Mosquera-Endara, 2022), whereas women are encouraged to be more expressive and emotionally dependent (Castillo-Gonzáles et al., 2026). “These norms may contribute to the observed gender differences in mental health outcomes.”

### 4.3 DaV and MH in women and men

Statistically significant associations were found between MH and DaV, consistent with findings from various authors (Betancourt Ocampo et al., 2022; Coker et al., 2002; Kaura and Lohman, 2007), who argue that DaV is related to MH problems, reflecting a relationship between victimization and a range of adverse psychological symptoms. These symptoms are commonly associated with anxiety, social dysfunction, depression, and somatic complaints.

Dimensions of detachment, humiliation, sexual, coercion and physical violence were related with poorer MH outcomes in participants, with this pattern being more pronounced in women than in men. Importantly, overall DaV, as well as sexual and coercion violence, showed stronger associations with MH problems in women. However, it is central to highlight than men who experience DaV also report mental health problems, although the intensity of these associations appears to be greater in women.

A possible explanation for these findings is that, although DaV is bidirectional between men and women, the severity and type of violence experienced differ by gender. In this regard, studies such as Castillo-Gonzáles et al. (2024) suggest that, while both men and women can be victims, men tend to report higher levels of mild forms of violence across different dimensions, whereas women experience more severe forms, particularly in terms of coercion (psychological violence) and sexual violence. This greater severity may help explain the stronger impact on mental health observed among women in the present study.

Additionally, differences in emotional socialization and patterns of distress expression may also contribute to these answers. Women are more likely to externalize their psychological distress, which may lead to greater identification and reporting of mental health symptoms (Bezerra et al., 2021). Moreover, various biological, psychological, and social factors have been associated with a higher predisposition among women to develop internalizing problems, such as anxiety and depression (Redondo Pacheco et al., 2022). In this context, involvement in violent romantic relationships may exacerbate these vulnerabilities, increasing psychological distress. But it is important to note that men who experience DaV also report mental health problems, although to a lesser extent. This result may be partly explained by gender norms that discourage emotional expression in men, potentially leading to an underestimation of their distress in self-report measures (Macassa et al., 2025).

### 4.4 Constraints

This document is not without limitations. First, the quantitative approach does not allow for a more detailed analysis of participants’ individual experiences or the contextualization of their behaviors. Also, the cross-sectional design of the study prevents establishing causal relationships between variables, limiting the findings to associative interpretations. Second, social desirability was not controlled in the responses, meaning that participants may have tended to respond in a way that was perceived to be favorable rather than responding accurately and honestly. This is particularly relevant in studies of relationship violence, as participants may feel uncomfortable or embarrassed to report violent or abusive behavior. Furthermore, the characteristics of the sample must be considered when interpreting the finding. The participants were primarily university students from urban areas and predominantly identified as mestizo, which may limit the generalizability of the results to other populations, such as persons from rural contexts or non-student groups. These limitations should be taken into account when interpreting the results of the study and planning future research.

## 5 Conclusion

This study found that DaV represents a serious issue that affects both women and men, with a slightly higher frequency reported among men. Although historically women have been identified as the primary victims of relationship violence, recent research (Díaz Burgos et al., 2023; Pacheco Maldonado and Castañeda Figueroa, 2017; Peña-Cárdenas et al., 2013; Rojas-Solís et al., 2019; Warthe et al., 2025) indicates that men also experience significant levels of violence. In Ecuador, DaV is influenced by social stigma, gender expectations, and limited availability of resources for victims (Cuadrado-Gordillo and Martín-Mora-Parra, 2022). It is essential to adopt an appropriate approach to DaV, including the development of interventions that empower individuals to recognize themselves as victims, report incidents, and exit violent relationship dynamics.

The investigation demonstrates that exposure to violence in intimate relationships is associated with a variety of MH issues, including somatization, depression, anxiety, and other psychological disorders. Thus, DaV has a significant negative impact on victims’ mental health. Furthermore, the data suggest that DaV is associated with poorer MH outcomes in women. Numerous studies (Kaura and Lohman, 2007; Lagdon et al., 2014; Lorenzo González et al., 2018) report that DaV is associated with of poorer MH in women.

The findings underscore the growing need for further research aimed at improving our understanding of DaV and MH in woman and men, as well as the development of targeted interventions for prevention and treatment. Moreover, both quantitative and qualitative research is necessary to explore risk and protective factors, analyze gendered experiences, and address the consequences of DaV on mental health from both male and female perspectives. Finally, these findings highlight the importance of gender-sensitive approaches in both prevention and intervention strategies.

## Data Availability

The original contributions presented in the study are included in the article/supplementary material, further inquiries can be directed to the corresponding author.
